# Hollow Ni/NiO/C composite derived from metal-organic frameworks as a high-efficiency electrocatalyst for the hydrogen evolution reaction

**DOI:** 10.1186/s40580-023-00354-w

**Published:** 2023-02-02

**Authors:** Ha Huu Do, Mahider Asmare Tekalgne, Quyet Van Le, Jin Hyuk Cho, Sang Hyun Ahn, Soo Young Kim

**Affiliations:** 1 School of Chemical Engineering and Materials Science, Chung-Ang University, 84 Heukseok-Ro, Dongjak-Gu, Seoul, 06974 Republic of Korea; 2 Department of Materials Science and Engineering, Institute of Green Manufacturing Technology, Korea University, 145 Anam-Ro, Seongbuk-Gu, Seoul, 02841 Republic of Korea

**Keywords:** MOFs, Ni/NiO/C, Hollow structure, Electrocatalyst, HER

## Abstract

Metal-organic frameworks (MOFs) constitute a class of crystalline porous materials employed in storage and energy conversion applications. MOFs possess characteristics that render them ideal in the preparation of electrocatalysts, and exhibit excellent performance for the hydrogen evolution reaction (HER). Herein, H–Ni/NiO/C catalysts were synthesized from a Ni-based MOF hollow structure via a two-step process involving carbonization and oxidation. Interestingly, the performance of the H–Ni/NiO/C catalyst was superior to those of H–Ni/C, H–NiO/C, and NH–Ni/NiO/C catalysts for the HER. Notably, H–Ni/NiO/C exhibited the best electrocatalytic activity for the HER, with a low overpotential of 87 mV for 10 mA cm^−2^ and a Tafel slope of 91.7 mV dec^−1^. The high performance is ascribed to the synergistic effect of the metal/metal oxide and hollow architecture, which is favorable for breaking the H–OH bond, forming hydrogen atoms, and enabling charge transport. These results indicate that the employed approach is promising for fabricating cost-effective catalysts for hydrogen production in alkaline media.

**Supplementary Information:**

The online version contains supplementary material available at 10.1186/s40580-023-00354-w.

## Introduction

The discovery of green energy sources has contributed significantly to the implementation of sustainable development practices in society, necessitated by the rising concerns associated with the negative impacts of fossil fuels on the environment. Hydrogen (H_2_) has been evaluated as a potential replacement for fossil fuels owing to its energy efficiency and eco-friendly properties [1–5]. Among the available methods for hydrogen production, water splitting exhibits promise. Typically, Pt-based materials exhibit excellent catalytic activity for water dissociation [6–8]. However, their lack of stability and high cost hinder their utilization on an industrial scale. Accordingly, numerous studies have explored the use of non-noble metal catalysts, such as transition metal selenides [9–11], metal nitrides [12–14], and metal carbides [15–17], which exhibit promising activity in acidic solutions [18–22]. However, their performance is still limited for the hydrogen evolution reaction (HER) in alkaline environments. This is because extra energy must be supplied for the Volmer process. To address this issue, many studies related to the use of Ni/NiO-based electrocatalysts for the HER under alkaline conditions have been conducted. Because Ni metal has relatively appropriate Gibbs free energy of adsorbed atomic hydrogen (‒ 0.27 eV) for HER [23]. Moreover, Ni is a non-noble metal, implying the synthesis of low-cost electrocatalysts from Ni-based materials [24]. On the other hand, NiO is effective for the dissociation of water molecules due to its defects [25]. For example, Oshchepkov et al. demonstrated the role of NiO in enhancing the HER performance of a Ni catalyst [26]. Yan et al. created a core–shell Ni/NiO architecture with outstanding durability [27]. Wang et al. recognized that the use of an appropriate Ni/NiO ratio results in a low overvoltage [28]. Yang et al. deposited carbon nanotubes on Ni/NiO nanofibers to achieve a high HER efficiency with a low overvoltage [29]. Overall, metal oxides play a vital role in the Volmer step, leading to improved HER activity [30–34]. They act as absorbents and scavenge hydroxyl ions, blocking the active metal sites.

Hollow-structured materials have also been considered as potential electrocatalysts for the HER. Hollow structures possess thin shells with inner and outer surfaces to afford a larger surface area and more exposed active sites that promote reactant adsorption and redox reactions, providing these surfaces with locally incompatible cofactors for oxidative and reductive reactions [35, 36]. In addition, hollow structures allow a shortened path for charge transport during the catalytic process and facilitate the growth of the other active materials [37–39]. For example, Nguyen et al. found that WS_2_ hollow spheres exhibited a higher HER catalytic activity than WS_2_ with a non-hollow structure [40]. Ganesan and Kim designed CoS_2_-MoS_2_ with a hollow architecture as a high-efficiency catalyst for the HER [41]. The effectiveness of the hollow structure was reported by Xia [42], who highlighted that CoP@MoS_2_ hollow nanocatalysts with a small Tafel slope are promising for the HER. Furthermore, Metal-organic frameworks (MOFs) are ideal for the synthesis of hollow-structured catalysts because of the level of morphological control they provide via changing the linkers and reaction conditions. In addition, MOFs possess a high porosity, which increases the active site density. Xue et al. employed ZIF-67 as a template to prepare CoSe_2_–FeSe_2_ catalysts with a hollow architecture for the HER under alkaline conditions [43]. Tian et al. used a Ni-MOF to create NiS_2_ hollow microspheres as highly stable catalysts for the HER [44]. As mentioned above, the use of Ni/NiO with a hollow structure derived from the MOF may lead to remarkable HER performance.

In this study, we used a Ni-MOF hollow structure to prepare a Ni/NiO/C hollow-structured composite through the two-step route of carbonization and oxidation. This material showed enhanced HER activity compared with Ni/C, NiO/C, and Ni/NiO/C with a non-hollow structure. Moreover, it exhibited a very low overpotential of 87 mV at a current density of 10 mA cm^−2^ and good durability. The approach employed herein could serve as a novel route for the synthesis of hollow-structured catalysts for the HER and other applications.

## Materials and methods

### Chemicals and materials

Nickel nitrate hexahydrate (Ni(NO_3_)_2_·6H_2_O), benzene-1,3,5-tricarboxylic acid (H_3_BTC, 95%), N, N-dimethylformamide (DMF), and polyvinylpyrrolidone (PVP, Mw = 40,000) and ethanol (C_2_H_5_OH) were purchased from Sigma Aldrich. Deionized water (DI) was prepared using a Millipore Milli-Q apparatus.

### Fabrication of the Ni-MOF hollow structure

Typically, a Ni-MOF hollow structure was synthesized according to a reported study with a slight modification [45]. Ni(NO_3_)_2_·6H_2_O (216 mg) and H_3_BTC (75 mg) were dissolved in a mixed solvent containing 5 mL of DMF, 5 mL of C_2_H_5_OH, and 5 mL of DI for 30 min under magnetic stirring. Subsequently, 750 mg of PVP, which acted as a micelle stabilizer, was added to this mixture under magnetic stirring for 2 h to form a homogeneous solution. This solution was transferred into a 25 mL Teflon-lined stainless-steel reactor and heated to 150 °C for 10 h. The obtained light-green Ni-MOF crystals were washed by C_2_H_5_OH and dried at 60 °C for 10 h.

### Fabrication of hollow-structured composites

The Ni-MOF powders were placed into an alumina crucible and heated at 450 °C for 2 h under a N_2_ gas to form the hollow architecture of H–Ni/C. Subsequently, this powder was oxidized in air at 250 °C for 20 min to obtain the H–Ni/NiO/C composite. H–NiO/C was obtained by heating the Ni-MOF hollow structure at 450 °C for 2 h. In addition, a non-hollow structure of NH–Ni/NiO/C was fabricated from a Ni-MOF non-hollow structure in the same manner as for the synthesis of the H–Ni/NiO/C composite.

### Material characterization

The morphological structure of the as-synthesized products was confirmed using scanning electron microscopy (SEM, Carl Zeiss) and transmission electron microscopy (TEM, JEOL). X-ray diffraction (XRD) tests were performed with a Bruker D8-Advance diffractometer. Raman curves of the composites were tested on a LabRAM-HR Evolution spectrometer with a 532 nm laser. The elemental composition was determined using X-ray photoelectron spectroscopy (XPS, K-alpha).

### Electrochemical measurements

The HER performance was evaluated using various electrochemical techniques with the help of an Ivium 55,630 instrument. The electrochemical system contained a graphite rod as the counter electrode, Hg/HgO electrode as the reference electrode, and 1 M KOH as the electrolyte. The working electrode was fabricated as follows: the as-synthesized materials (30 mg) were sonicated in a mixture containing 0.1 mL Nafion (5 wt%) and 3 mL C_2_H_5_OH for 30 min. Subsequently, a certain volume of the homogeneous suspension was coated on the surface of a glassy carbon electrode (diameter of 3 mm), with a catalyst loading of 0.30 mg cm^−2^. Polarization curves were collected with IR correction at a scan rate of 2 mV s^−1^. IR corrections for resistance were conducted using electrochemical impedance spectroscopy (EIS) in the potentiostatic mode from 100,000 to 0.1 Hz. Cyclic voltammetry (CV) was performed to determine the electrochemical double-layer capacitance (C_dl_) in the non-faradaic voltage region at 20, 40, 60, 80, 120, and 140 mV s^−1^. The reported potentials were converted to those of the reversible hydrogen electrode (RHE) using the following relation: E_RHE_ = E_Hg/HgO_ + 0.098 + 0.059pH.

## Results and discussion

**Fig. 1:**
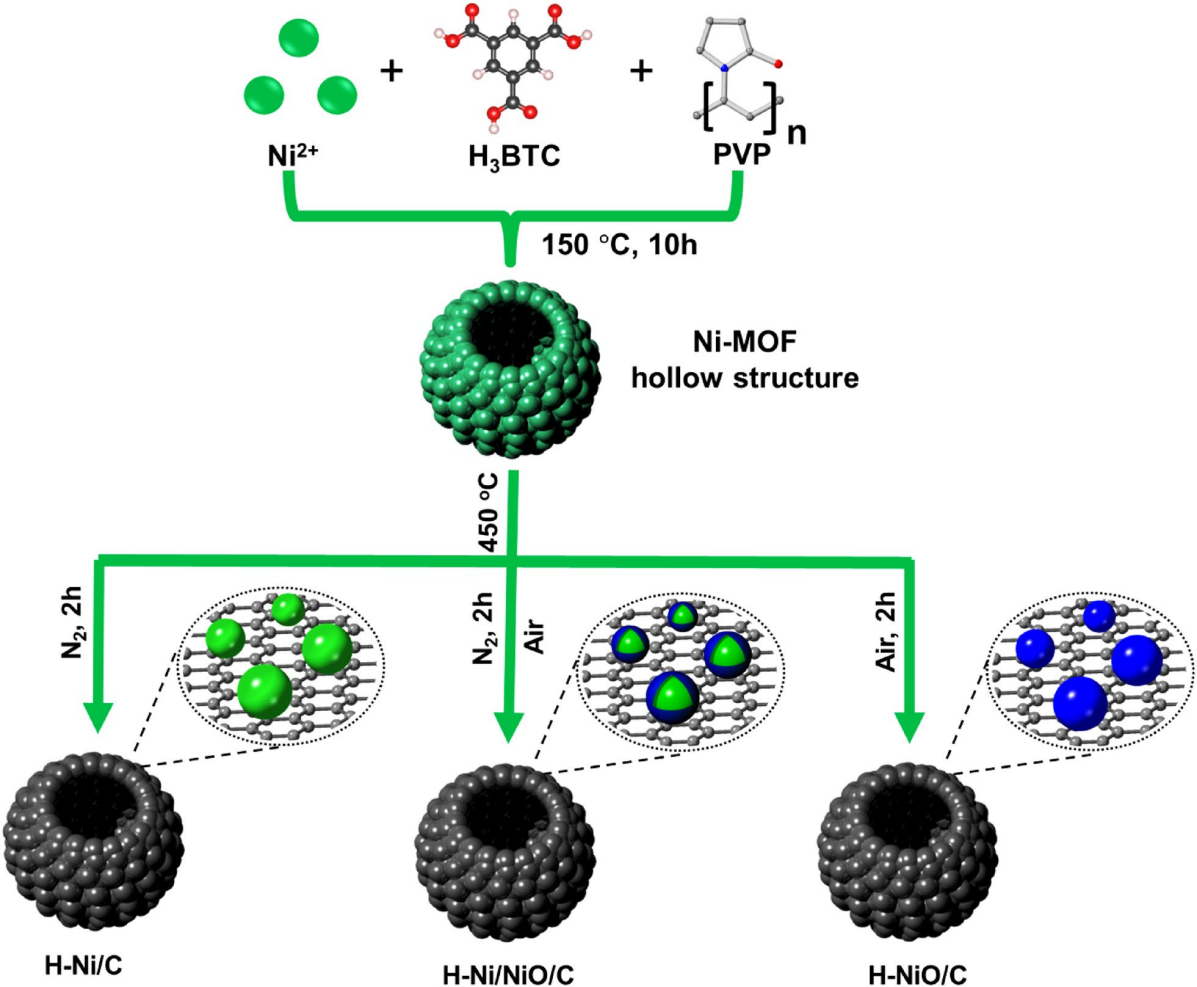
Schematic illustrating the fabrication of H–Ni/C, H–Ni/NiO/C, and H–NiO/C catalysts

**Fig. 2:**
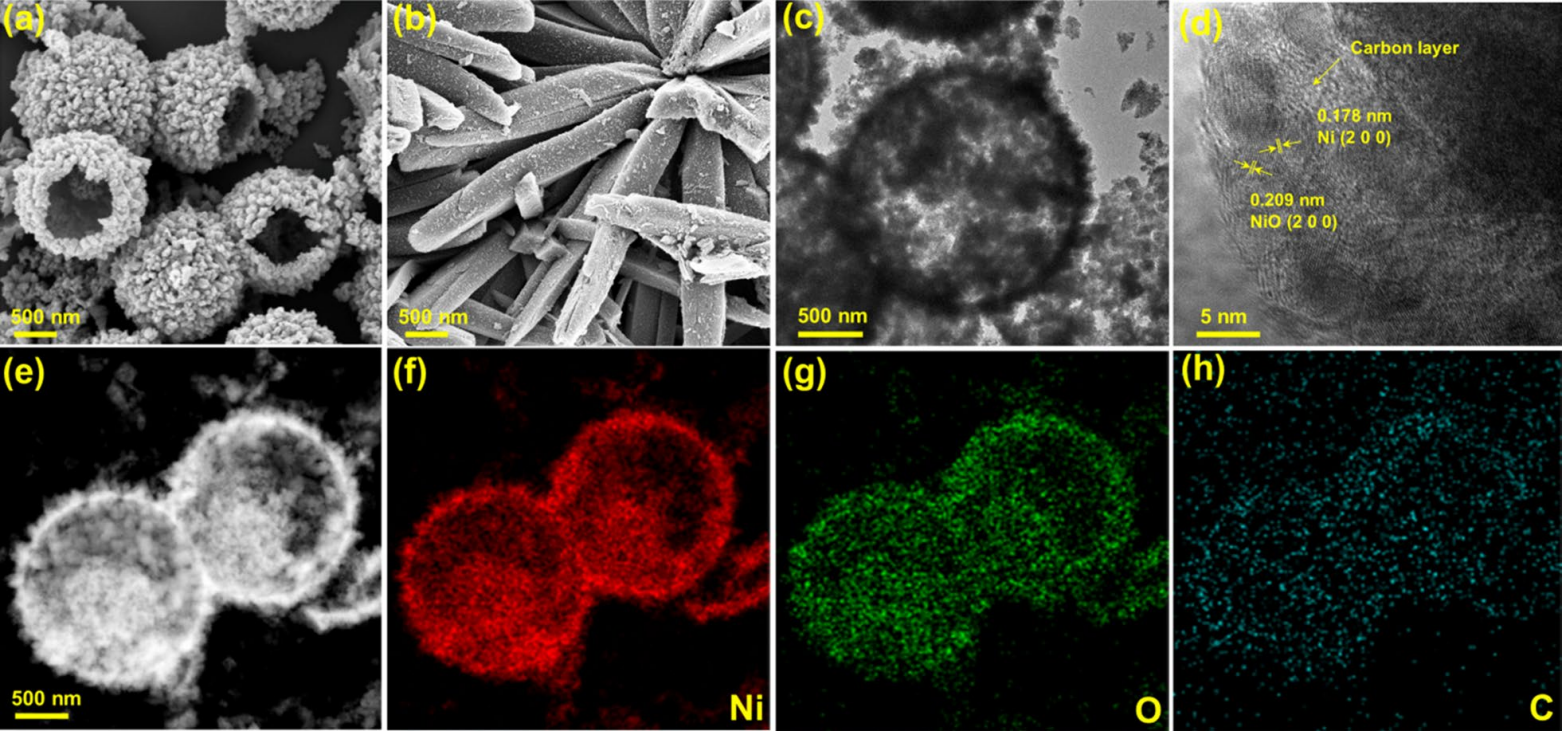
SEM images of **a** H–Ni/NiO/C and **b** NH–Ni/NiO/C, **c** TEM, **d** HR-TEM, **e** Scanning TEM images of H–Ni/NiO/C with corresponding element maps of **f** Ni, **g** O, and **h** C

Herein, we used a Ni-MOF hollow structure to fabricate H–Ni/NiO/C via a two-step process involving pyrolysis and oxidation, as schematically shown in Fig. 1. The Ni-MOF hollow precursor was synthesized through a typical synthetic route [46, 47]. Subsequently, the as-synthesized Ni-MOF hollow precursor was carbonization for 2 h to prepare H–Ni/C, followed by oxidation in the air for various durations to form H–Ni/NiO/C. In this step, the Ni species that were embedded in the carbon skeleton partially oxidized into NiO phases. Moreover, H–NiO/C was generated by the direct calcination of the Ni-MOF precursor at 450 °C in the air for 2 h. The phase structures and microtopographies of the Ni-MOF and its derivatives were analyzed using XRD and SEM. The XRD patterns in Additional file 1: Fig. S1a show a main peak at 13.0$^\circ$ ascribed to the Ni-MOF, indicating the existence of an organic composition [45, 47–49]. The Ni-MOF crystals exhibit a spherical morphology with an average size of 1.5 µm. The hollow structure of the Ni-MOF was confirmed by SEM (Additional file 1: Fig. S1b). The surface of the microspheres was not smooth, implying that they were assembled from nanoparticles. As depicted in the SEM images (Fig. 2a and Additional file 1: Fig. S2), the H–Ni/C, H–Ni/NiO/C, and H–NiO/C spheres possess a well-preserved hollow architecture similar to that of the hollow Ni-MOF precursor. This indicates that the carbon skeleton does not collapse during pyrolysis and oxidation. In addition, the SEM image in Fig. 2b confirms the synthesis of Ni/NiO/C with a non-hollow structure and an average size of 0.5 × 3 µm. The TEM image (Fig. 2c) verifies that the H–Ni/NiO/C samples possess a hollow morphological structure with a particle diameter of 1.4 µm. The high-resolution TEM image indicates that the Ni/NiO nanograins are embedded in the carbon matrix (Fig. 2d). The d-spacing of 0.178 nm was indexed to the (200) crystal plane of metallic Ni, whereas that of 0.209 nm is assigned to the (200) crystal plane of the NiO phase. Moreover, (111) crystal plane of H–Ni/C with a d-spacing of 0.202 nm is indicated in Additional file 1: Fig. S3. As depicted in Fig. 2e–h, scanning TEM and a corresponding elemental mapping of H–Ni/NiO/C indicate the well-distribution of elements (Ni, O, C).

**Fig. 3:**
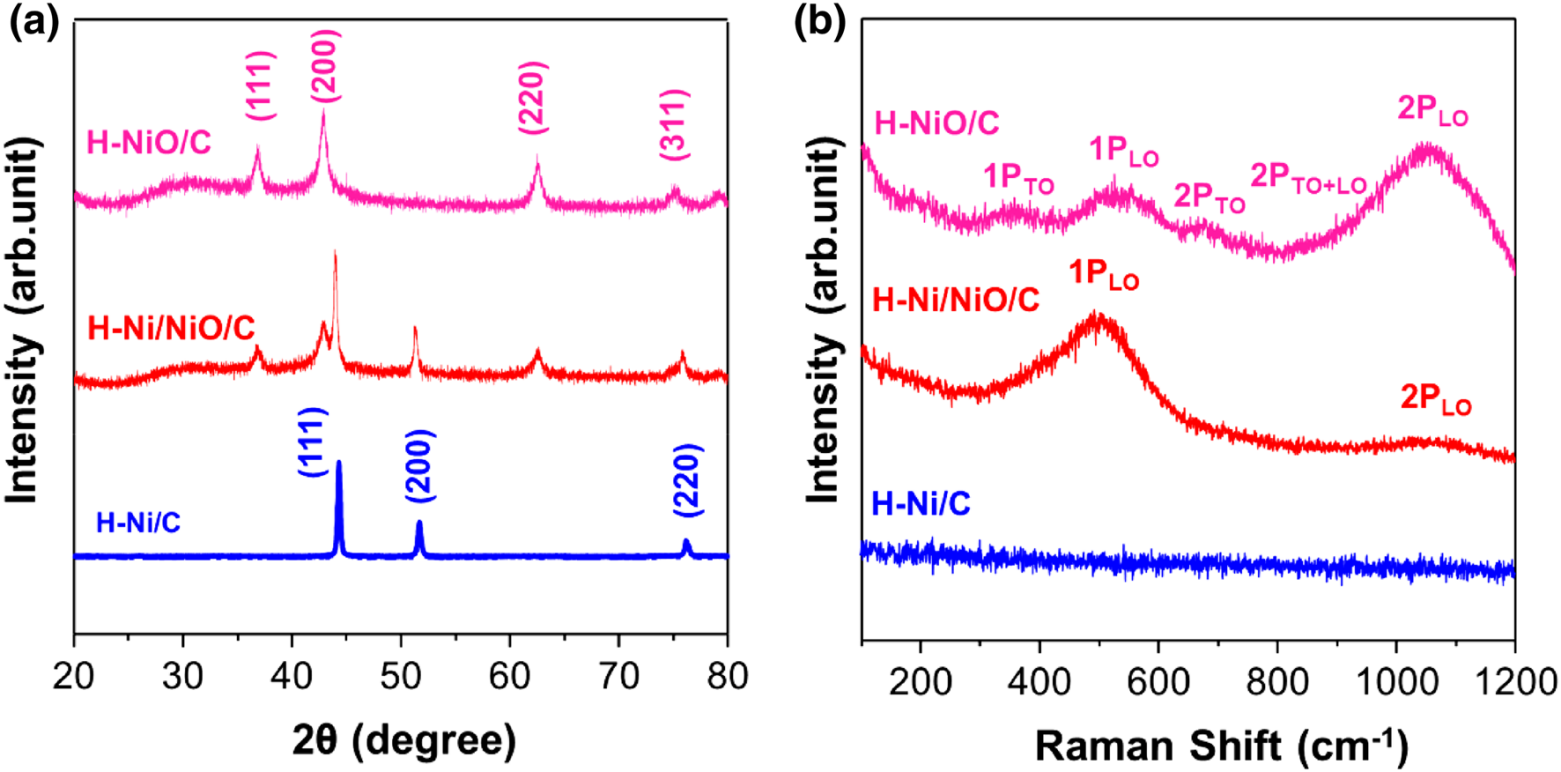
**a** XRD patterns and **b** Raman spectrum of H–Ni/C, H–Ni/NiO/C, and H–NiO/C

Figure 3a shows the XRD patterns of H–Ni/C, H–Ni/NiO/C, and H–NiO/C. The peaks at 2θ values of 44.3°, 51.5°, and 76.1° can be indexed to the (111), (200), and (220) planes of metallic Ni (PDF 00-001-1258), respectively. The XRD pattern of H–NiO/C shows peaks at 37.2°, 43.2°, and 62.9°, which are assigned to the (111), (200), and (220) planes (PDF 00-004-0835), respectively [50, 51]. The Raman spectra of the H–Ni/C, H–Ni/NiO/C, and H–NiO/C catalysts are shown in Fig. 3b. The H–Ni/C sample is not Raman-active because there is no observable change in the polarizability of metallic Ni [52]. The spectrum for H–NiO/C displays five peaks at 360, 510, 680, 840, and 1060 cm^−1^, whereas that for H–Ni/NiO/C exhibits two peaks at 500 and 1060 cm^−1^. The Raman peaks at 360 and 500–510 cm^−1^ can be indexed to the one-phonon (1P) transverse optical (TO) and longitudinal optical (LO) vibrations of the Ni–O bonds. The other peaks are assigned to the second-order vibrational modes of 2P_TO_, 2P_TO+LO_, and 2P_LO_, respectively [53, 54]. Interestingly, the intensity of 1P_LO_ increased, whereas the intensity of 2P_LO_ decreased in the H–Ni/NiO/C. Because the intensity of 1P_LO_ and 2P_LO_ was related to the defect states associated with Ni vacancies [55, 56]. According to Mishra et al., the increased temperature reduces the Ni vacancies [57], which dominates the intensity of 1P_LO_ [58]. Therefore, the high intensity of 1P_LO_ could be ascribed to a large number of Ni vacancies in H–Ni/NiO/C, which was formed at 250 °C. H–NiO/C created at 450 °C shows a low intensity of 1P_LO_ mode, which was attributed to the decelerated Ni vacancies. Also, the intensity of 2P_LO_ was accelerated due to the improved crystalline quality of the H–NiO/C sample [59].

**Fig. 4:**
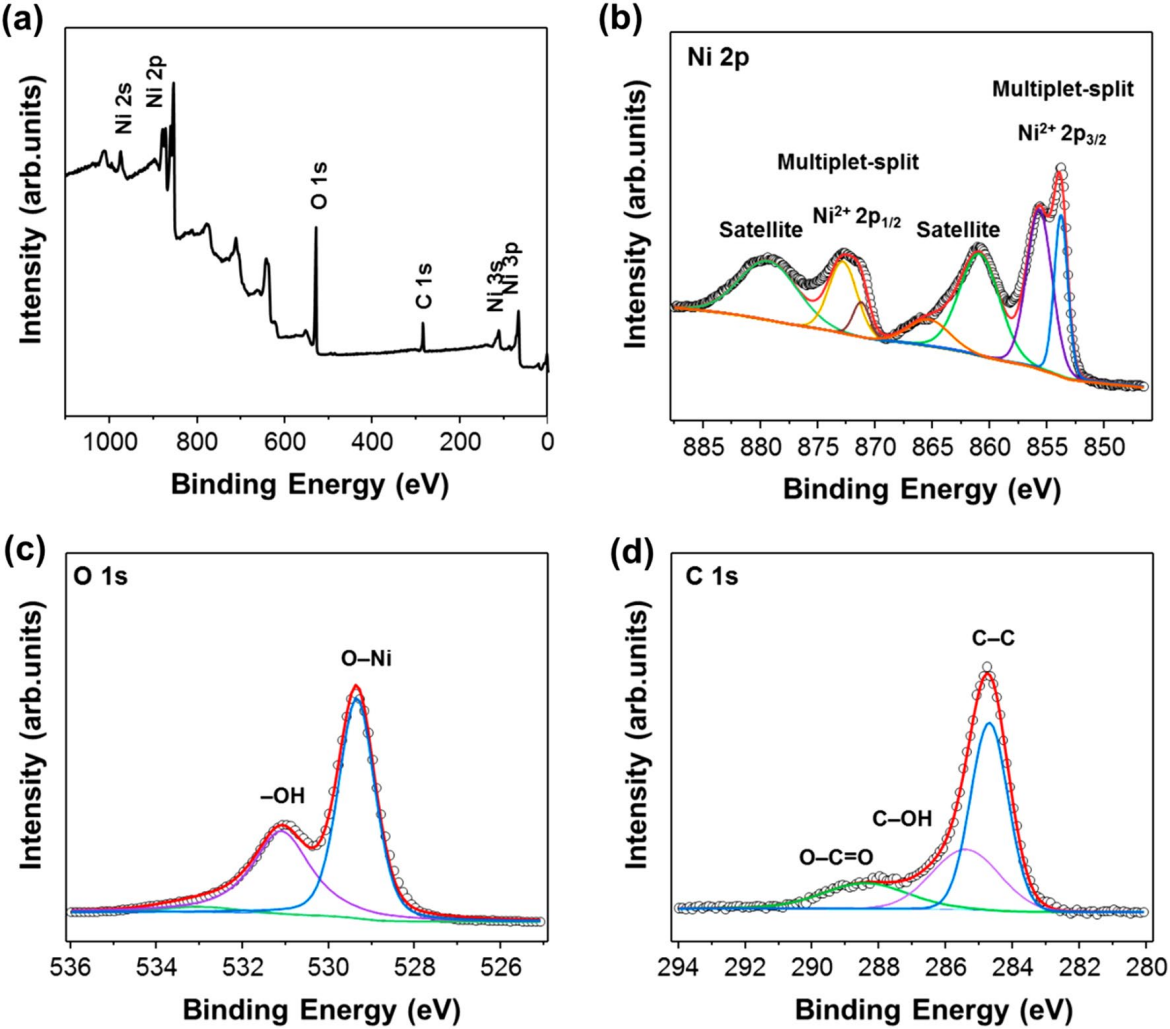
**a** XPS survey spectrum. High-resolution XPS profiles of H–Ni/NiO/C: **b** Ni 2p, **c** O 1 s, and **d** C 1 s

The valence states of Ni, C, O were confirmed by XPS. Peaks of these elementals are clearly observed in the total XPS profiles of H–Ni/NiO/C in Fig. 4a. Notably, the HR XPS profiles of Ni 2p are deconvoluted into two main peaks, Ni^2+^ 2p_1/2_ and Ni^2+^ 2p_3/2_, as depicted in Fig. 4b. Notably, a multiplet-split Ni^2+^ 2p_3/2_ at 853.7 and 855.5 eV, and a Ni^2+^ 2p_3/2_ satellite at 860.8 eV. Also, the peaks at 870.9 eV and 872.4 eV correspond to the multiplet-split Ni^2+^ 2p_1/2_ and Ni^2+^ 2p_3/2_ of NiO phases, respectively [27]. This is also confirmed in the XPS spectrum of Ni 2p for the H–NiO/C sample (Additional file 1: Fig. S4). More importantly, Peaks at 852.6 eV for metallic Ni 2p_3/2_ and 869.9 eV for metallic Ni 2p_1/2_ (Additional file 1: Fig. S5) do not appear in the XPS spectrum of H–Ni/NiO/C sample, whereas XRD patterns confirmed metallic Ni in H–Ni/NiO/C [51]. This outcome was attributed to the formation of NiO on the surface metallic Ni and the difference in depth penetration in the sample of the two methodologies [60]. Figure 4c shows the O1s spectra of H–Ni/NiO/C; the two characteristic peaks are attributed to Ni–O (529.3 eV) and – OH/oxygen vacancies (531.1 eV) [27]. The C1s spectra of H–Ni/NiO/C exhibit three types of contributions. The peaks at binding energies of 284.7, 285.4, and 288.4 eV can be indexed to the C‒C, C‒OH, and O‒C = O bonds, respectively [61]. Besides, the Ni:O:C atomic ratio measured from XPS is equal to 34.76:43.72:21.52. Ni concentration (34.76%) is smaller than the concentration of oxygen (43.72%), which implies the high Ni vacancies in the H–Ni/NiO sample. This is consistent with the Raman spectra of the H–Ni/NiO/C sample. Rich Ni vacancies in H–Ni/NiO/C can illustrate abundant active centers for HER [58].

**Fig. 5:**
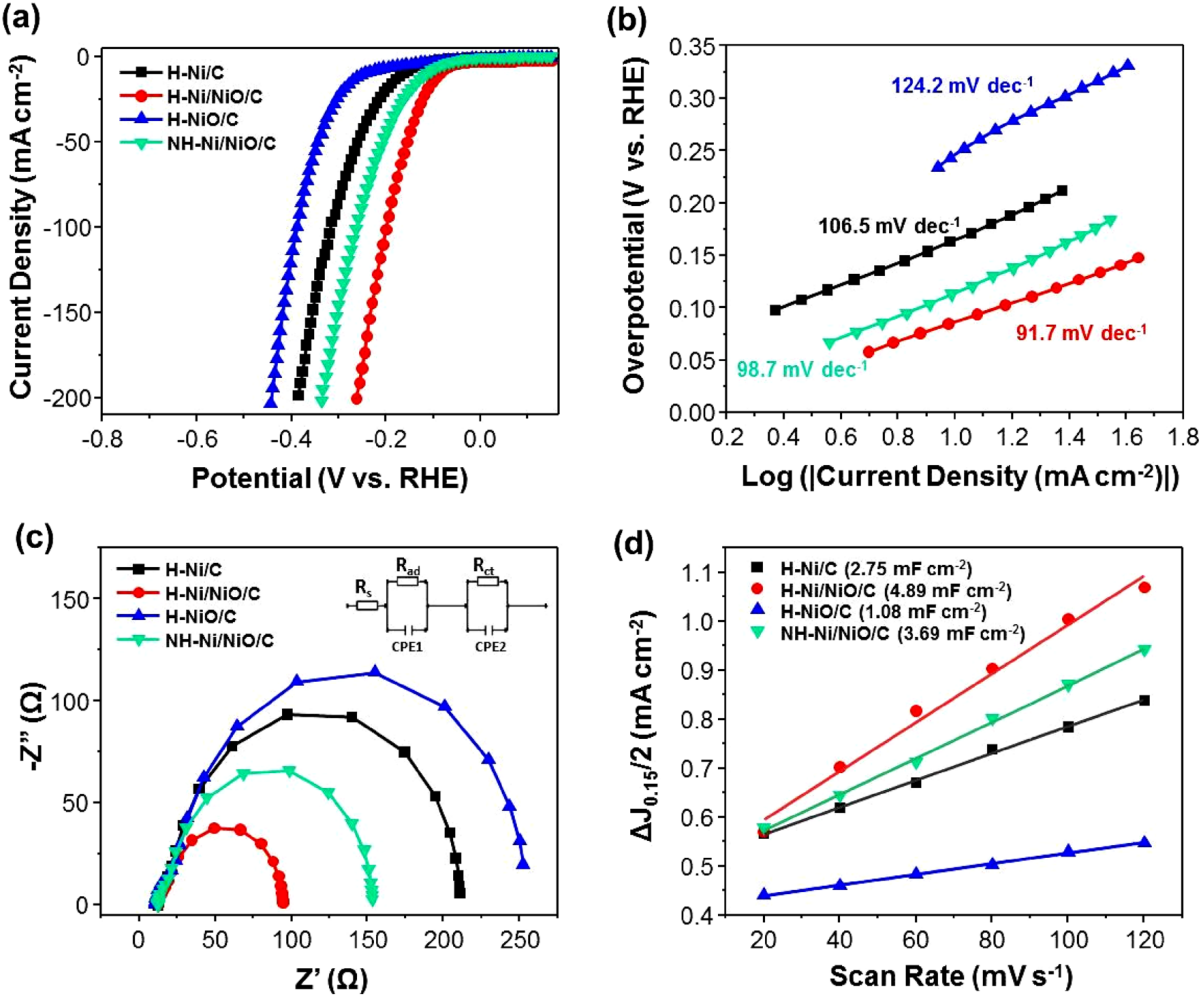
**a** Polarization curve of H–Ni/C, H–Ni/NiO/C, H–NiO/C, and NH–Ni/NiO/C. **b** Tafel slope of catalysts, **c** Nyquist plots of different samples recorded at a potential of − 0.2 V vs. the RHE. **d** Double-layer capacitances of various samples obtained using CV

The HER performances of H–Ni/C, H–Ni/NiO/C, H–NiO, and NH–Ni/NiO/C were investigated in a 1.0 M potassium hydroxide medium. As displayed in Fig. 5a, H-Ni/NiO/C exhibits a higher catalytic activity than H–Ni/C, H–NiO, and NH–Ni/NiO/C (Additional file 1: Fig. S6). To achieve an HER current density of 10 mA cm^−2^, the H–Ni/C, H–Ni/NiO/C, H–NiO, and NH–Ni/NiO/C electrocatalysts require overpotentials of 167, 87, 246, and 117 mV, respectively (Additional file 1: Table S1). A lower overvoltage indicates a lower energy barrier requirement for the HER. During the HER with the H–Ni/NiO/C electrocatalyst, the NiO species plays an important role in breaking the H–OH bond to generate H atoms, which then traverse to the Ni species before forming H_2_ molecules [23, 25, 62, 63]. When using the H–Ni/C electrocatalyst, the active sites are impeded by the OH- ions generated from water dissociation [52, 64]. Moreover, the hollow materials provide more exposed active centers than their bulk counterparts and enable electron transfer during the catalytic process [42, 65, 66]. Therefore, the hollow structure of H–Ni/NiO/C exhibits a higher HER activity than that of the non-hollow structure of NH–Ni/NiO/C. Also, H–Ni/NiO/C originated from Ni-MOF hollow structure exhibits a lower overpotential than the other electrocatalysts, which were not synthesized from MOFs precursors (Additional file 1: Table S2). Furthermore, using MOFs can easily control the morphology of electrocatalysts by changing reaction conditions. This outcome proved the advantage of MOFs for the preparation of electrocatalysts for HER. In addition, the Tafel plots show that the HER performance of H–Ni/NiO/C is superior to those of the other catalysts (Fig. 5b). In particular, the Tafel slopes of H–Ni/C, H–Ni/NiO/C, H–NiO, and NH–Ni/NiO/C are 106.5, 91.7, 124.2, and 98.7 mV dec^−1^, respectively. Typically, the HER in an alkaline solution involves three basic reactions, as follows:

$${\text{Catalyst}}\, + \,{\text{H}}_{{2}} {\text{O}}\, + \,{\text{e}}^{ - } \, \to \,{\text{Catalyst}} - {\text{H}}_{{{\text{ads}}}} \, + \,{\text{OH}}^{ - } ({\text{Volmer reaction}})$$

$${\text{Catalyst}} - {\text{H}}_{{{\text{ads}}}} \, + \,{\text{H}}_{{2}} {\text{O}}\, + \,{\text{e}}^{ - } \, \to \,{\text{Catalyst}}\, + \,{\text{H}}_{{2}} \, + \,{\text{OH}}^{ - } ({\text{Heyrovsky reaction}})$$

$${\text{Catalyst}} - {\text{H}}_{{{\text{ads}}}} \, + \,{\text{Catalyst}} - {\text{H}}_{{{\text{ads}}}} \, \to \,{\text{H}}_{{2}} \, + \,{\text{Catalyst }}({\text{Tafel reaction}})$$

In the Volmer reaction (Tafel slope ≈ 120 mV dec^−1^), the H–OH bond breaks to form H atoms that are absorbed on the surface of the catalyst. The Heyrovsky reaction (Tafel slope ≈ 40 mV dec^−1^) describes electrochemical desorption, whereas the Tafel reaction (Tafel slope ≈ 30 mV dec^−1^) expresses chemical desorption to form hydrogen. Based on the reported Tafel slopes, the Volmer and Heyrovsky reactions occur when using the investigated catalysts.

**Fig. 6:**
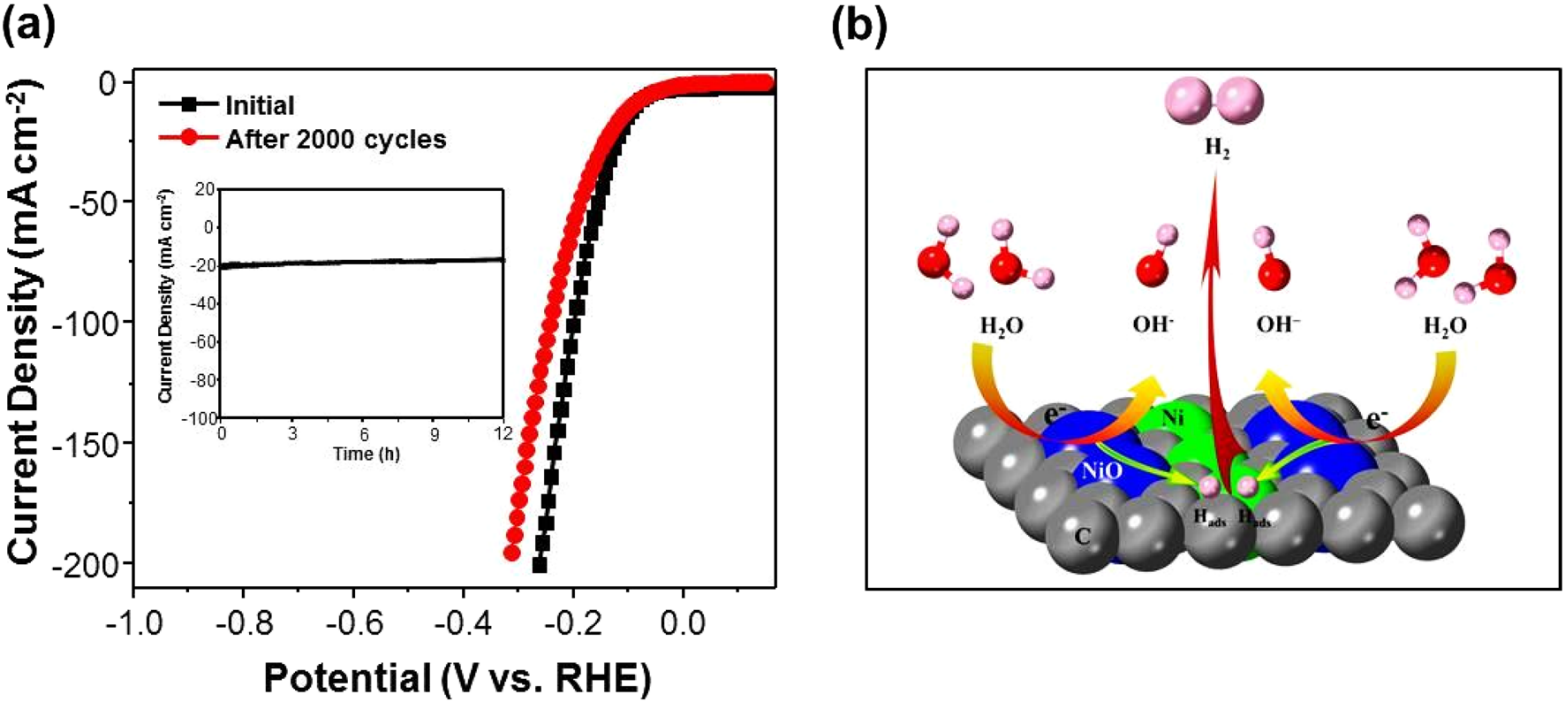
**a** Polarization curves recorded initially and after 2000 CV cycles of H–Ni/NiO/C. Inset: Current–time response of H–Ni/NiO/C obtained at − 0.198 V for 12 h. **b** Schematic of the proposed HER mechanism using the H–Ni/NiO/C catalyst

EIS was performed on the catalysts to confirm the reaction kinetics of the electrodes. Figure 5c shows the Nyquist plots of the four samples, which are fitted with an equivalent circle (inset of Fig. 5c) to determine the charge transfer resistance (R_ct_). Note that the R_ct_ of H–Ni/NiO/C is 75 Ω, which is much lower than that of H–Ni/C (189 Ω), H–NiO (229.4 Ω), and NH–Ni/NiO/C (133.4 Ω). The lowest R_ct_ of H–Ni/NiO/C is indicative of the fastest interfacial charge transport kinetics of this catalyst during the electrocatalytic process. Typically, the electrochemical surface area (ECSA) can be predicted from C_dl_, which is derived from the CV profiles in the non-faradaic potential region. The CV profiles of H–Ni/C (Additional file 1: Fig. S7a), H–Ni/NiO/C (Additional file 1: Fig. S7b), H–NiO (Additional file 1: Fig. S7c), and NH–Ni/NiO/C (Additional file 1: Fig. S7d) were acquired in the potential window of 0.1 to 0.2 V (vs. the RHE) at various scan rates. The slope of the plot of ΔJ/2 at 0.15 V (vs the RHE) versus the scan rate is C_dl_. The C_dl_ of H–Ni/NiO/C is 4.89 mF cm^−2^, which is larger than those of H–Ni/C (2.75 mF cm^−2^), H–NiO (1.08 mF cm^−2^), and NH–Ni/NiO/C (3.69 mF cm^−2^), implying its larger ECSA as well as superior HER performance (Fig. 5d). The durability of H–Ni/NiO/C was evaluated using the CV profiles and the constant potential method. The results are shown in Fig. 6a. The linear sweep voltammetry (LSV) of H–Ni/NiO/C is only slightly altered compared to the initial curve. In particular, the overvoltage at 10 mA cm^−2^ is increased only by 5 mV after 2000 cycles. The constant potential results reveal that nearly 90% of the initial current density is maintained after 12 h of operation. In addition, the crystal phase and morphological structure of H–Ni/NiO/C were confirmed by XRD and SEM after stability testing (Additional file 1: Fig. S8). The result revealed that H–Ni/NiO/C exhibited outstanding durability in alkaline environments. The mechanism of the HER using the H–Ni/NiO/C electrocatalyst is shown in Fig. 6b. In this mechanism, when water molecules are absorbed at the Ni/NiO interface, NiO moieties drive H–OH bond breakage to create adsorbed hydrogen atoms (H_ads_) and hydroxyl ions (OH^‒^), whereas Ni moieties recombine H_ads_ to produce H_2_. This synergistic effect helps H–Ni/NiO/C achieve a higher HER activity than bare electrocatalysts.

## Conclusion

In conclusion, Ni/NiO/C hollow-structured composites were successfully fabricated from Ni-MOF precursors and utilized as efficient electrocatalysts for the HER in alkaline media. The experimental results confirmed that H–Ni/NiO/C exhibited better HER activity than H–Ni/C, H–NiO/C, and non-hollow NH–Ni/NiO/C catalysts. Notably, H–Ni/NiO/C exhibited a very low overvoltage of 87 mV at a current density of 10 mA cm^−2^. In addition, this catalyst exhibited remarkable stability after 12 h test. The good HER activity was assigned to the emerging effect of the metal/metal oxide and hollow morphological structure, which facilitated H–OH bond breakage, the recombination of intermediate hydrogen, and electron transfer. The empirical findings of this study provide a novel understanding of the use of MOFs for the fabrication of economical catalysts for H_2_ production.

## Supplementary Information

**Additional file 1: Figure S1.** (a) XRD pattern and (b) FE-SEM image of the Ni-MOF hollow structure. **Figure S2. **SEM images of (a) H–Ni/C and (b) H–NiO/C. **Figure S3.** (a) TEM, and (b) Field emission TEM images of H–Ni/C. **Figure S4. **(a) XPS survey spectrum. High-resolution XPS profiles of H–NiO/C: (b) Ni 2p, (c) O 1s, and (d) C 1s. **Figure S5**. High-resolution XPS profiles of H–Ni/C: (a) Ni 2p, and (b) C 1s. **Figure S6.** (a) Polarization curves of the H–Ni/NiO/C-10min, H–Ni/NiO/C-20min, and H–Ni/NiO/C-30min catalysts at a scan rate of 2 mV s^-1^ in a 1 M KOH solution. (b) Tafel plots of the same catalysts derived from (a). **Figure S7.** Cyclic voltammograms (0.1–0.2 V) of (a) H–Ni/C, (b) H–Ni/NiO/C, (c) H–NiO/C, and (d) NH–Ni/NiO/C at various scan rates (20–120 mV s^-1^) in a 1 M KOH solution. **Figure S8.** (a) XRD pattern and (b) SEM images of H–Ni/NiO/C after 2000 CV cycles. **Table S1. **Comparison of the HER performance of different samples in an alkaline solution. **Table S2. **Comparison of the catalytic activity of the Ni/NiO/C hollow structure with those of nickel-based catalysts for the HER in an alkaline solution.

## Data Availability

The datasets used and/or analyzed during the current study are available from the corresponding author upon reasonable request.

## Abbreviations

H_2_: Hydrogen
HER: Hydrogen evolution reaction
TM: Transition metal
TMO: Transition metal oxide
MOFs: Metal-organic framework
SEM: Scanning electron microscopy
XRD: X-ray diffraction
XPS: X-ray photoelectron spectroscopy
CV: Cyclic voltammetry
EIS: Electrochemical impedance spectroscopy
TEM: Transmission electron microscopy
HR–TEM: High resolution–transmission electron microscopy
PVP: Polyvinylpyrrolidone
C_2_H_5_OH: Ethanol
DI: De-ionized
GCE: Glassy carbon electrode
LSV: Linear sweep voltammetry
RHE: Reference hydrogen electrode
ECSA: Electrochemical active surface area
C_dl_: Double-layer capacitance
