# Supplementary material for: Hollow Ni/NiO/C composite derived from metal-organic frameworks as a high-efficiency electrocatalyst for the hydrogen evolution reaction
Source: Nano Converg. 2023 Feb 2;10:6. doi: 10.1186/s40580-023-00354-w (PMC9895561; doi:10.1186/s40580-023-00354-w)
Supplement: Supplementary file 1 — Additional file 1: Figure S1. (a) XRD pattern and (b) FE-SEM image of the Ni-MOF hollow structure. Figure S2. SEM images of (a) H–Ni/C and (b) H–NiO/C. Figure S3. (a) TEM, and (b) Field emission TEM images of H–Ni/C. Figure S4. (a) XPS survey spectrum. High-resolution XPS profiles of H–NiO/C: (b) Ni 2p, (c) O 1s, and (d) C 1s. Figure S5. High-resolution XPS profiles of H–Ni/C: (a) Ni 2p, and (b) C 1s. Figure S6. (a) Polarization curves of the H–Ni/NiO/C-10min, H–Ni/NiO/C-20min, and H–Ni/NiO/C-30min catalysts at a scan rate of 2 mV s-1 in a 1 M KOH solution. (b) Tafel plots of the same catalysts derived from (a). Figure S7. Cyclic voltammograms (0.1–0.2 V) of (a) H–Ni/C, (b) H–Ni/NiO/C, (c) H–NiO/C, and (d) NH–Ni/NiO/C at various scan rates (20–120 mV s-1) in a 1 M KOH solution. Figure S8. (a) XRD pattern and (b) SEM images of H–Ni/NiO/C after 2000 CV cycles. Table S1. Comparison of the HER performance of different samples in an alkaline solution. Table S2. Comparison of the catalytic activity of the Ni/NiO/C hollow structure with those of nickel-based catalysts for the HER in an alkaline solution. [file 40580_2023_354_MOESM1_ESM.docx]

**Hollow Ni/NiO /C Composite Derived from Metal–Organic Frameworks as a High-Efficiency Electrocatalyst for the Hydrogen Evolution Reaction**

*Ha Huu Do ^a^, Mahider Asmare Tekalgne ^a^, Quyet Van Le ^b^, Jin Hyuk Cho ^b^, Sang Hyun Ahn ^a,*^, Soo Young Kim ^c,*^*

^a^ School of Chemical Engineering and Materials Science, Chung-Ang University, 84 Heukseok-ro, Dongjak-gu, Seoul 06974, Republic of Korea

^b^Department of Materials Science and Engineering, Institute of Green Manufacturing Technology, Korea University, 145 Anam-ro, Seongbuk-gu, Seoul 02841, Republic of Korea.

***Corresponding author**

[Email: shahn@cau.ac.kr](mailto:Email:%20shahn@cau.ac.kr) (S.H. Ahn);

Email: [sooyoungkim@korea.ac.kr](mailto:sooyoungkim@korea.ac.kr) (S.Y. Kim);

**Synthesis of Ni-MOF non hollow structure**

Ni(NO_3_)_2_.6H_2_O (216 mg) and H_3_BTC (75 mg) were dissolved in a mixed solvent containing 5 mL of DMF, 5 mL of C_2_H_5_OH, and 5 mL of DI for 30 min under magnetic stirring. This solution was transferred into a 25 mL Teflon-lined stainless-steel reactor and heated to 150$^{\circ}$C for 10 h. The obtained green Ni-MOF crystals were washed by C_2_H_5_OH and dried at 60$^{\circ}$C for 10 h.

**
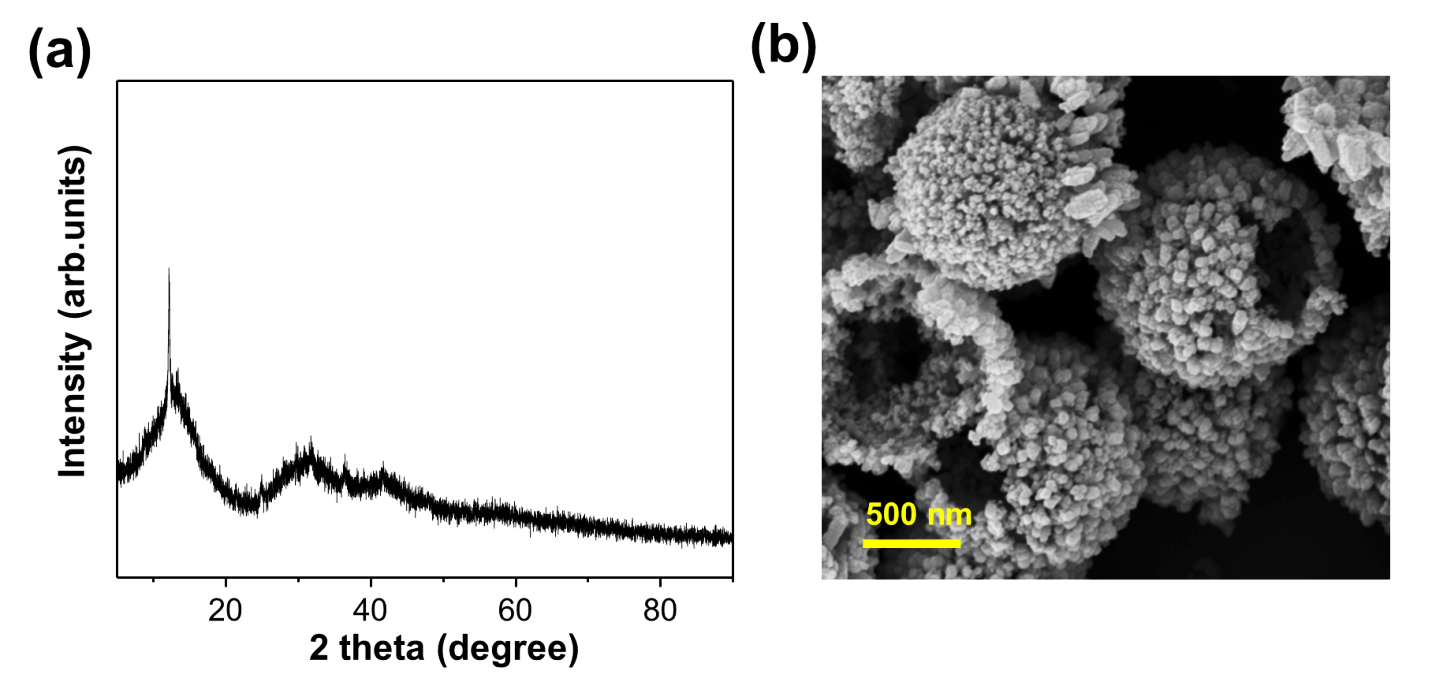
**

**Fig. S1**. (a) XRD pattern and (b) FE-SEM image of the Ni-MOF hollow structure


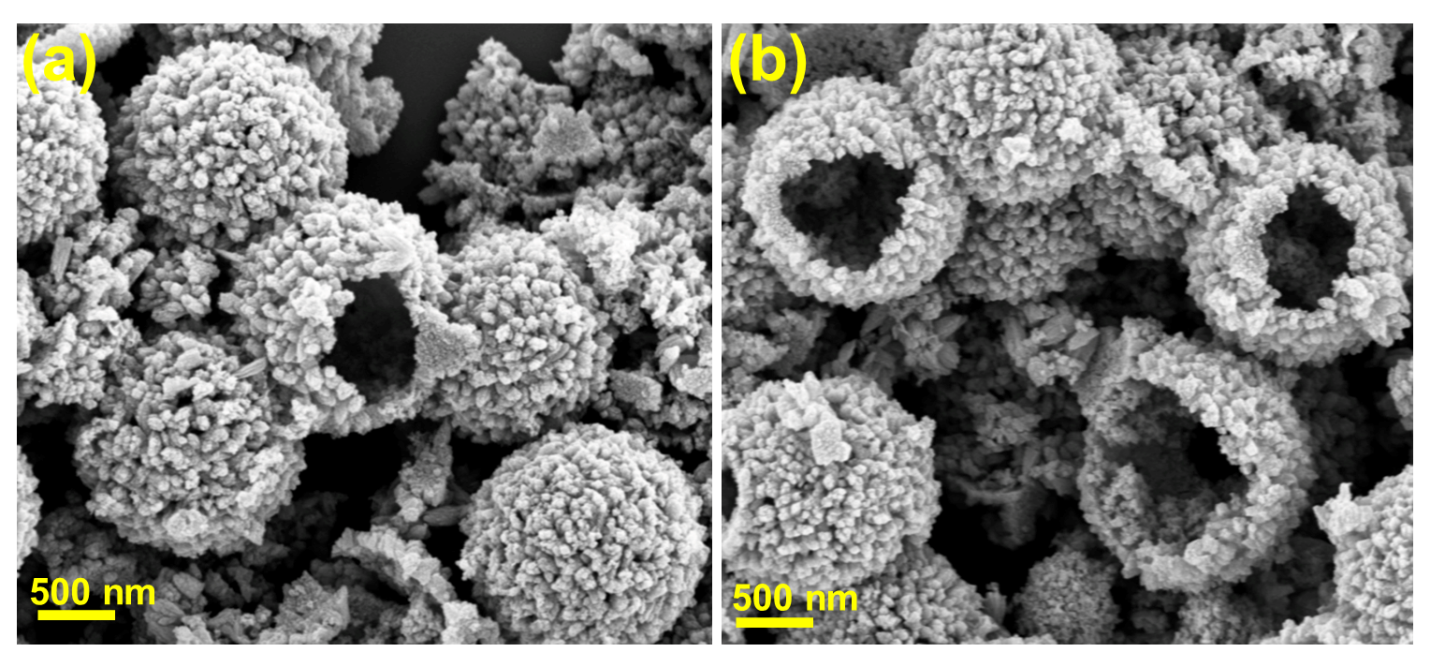


**Fig. S2**. SEM images of (a) H-Ni/C and (b) H-NiO/C

**
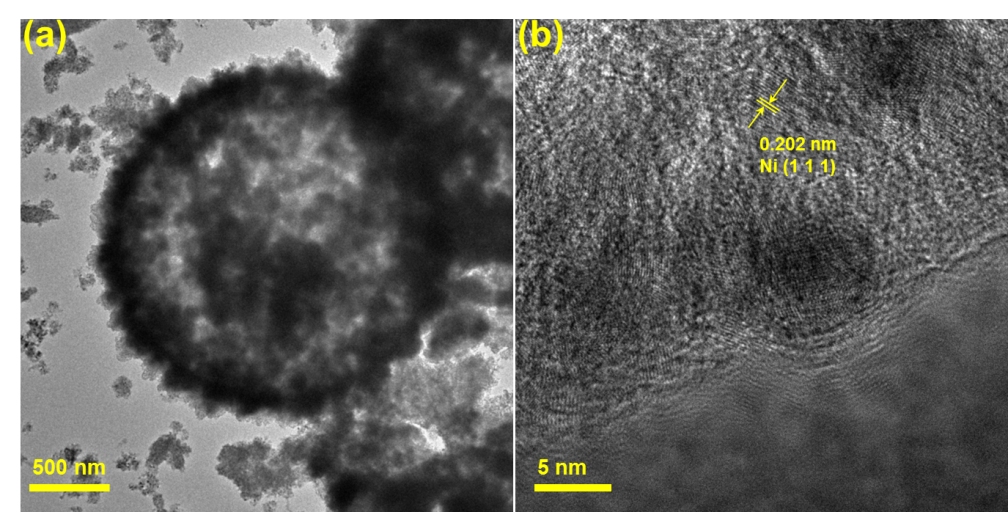
**

**Fig S3**. (a) TEM, and (b) Field emission TEM images of H-Ni/C.


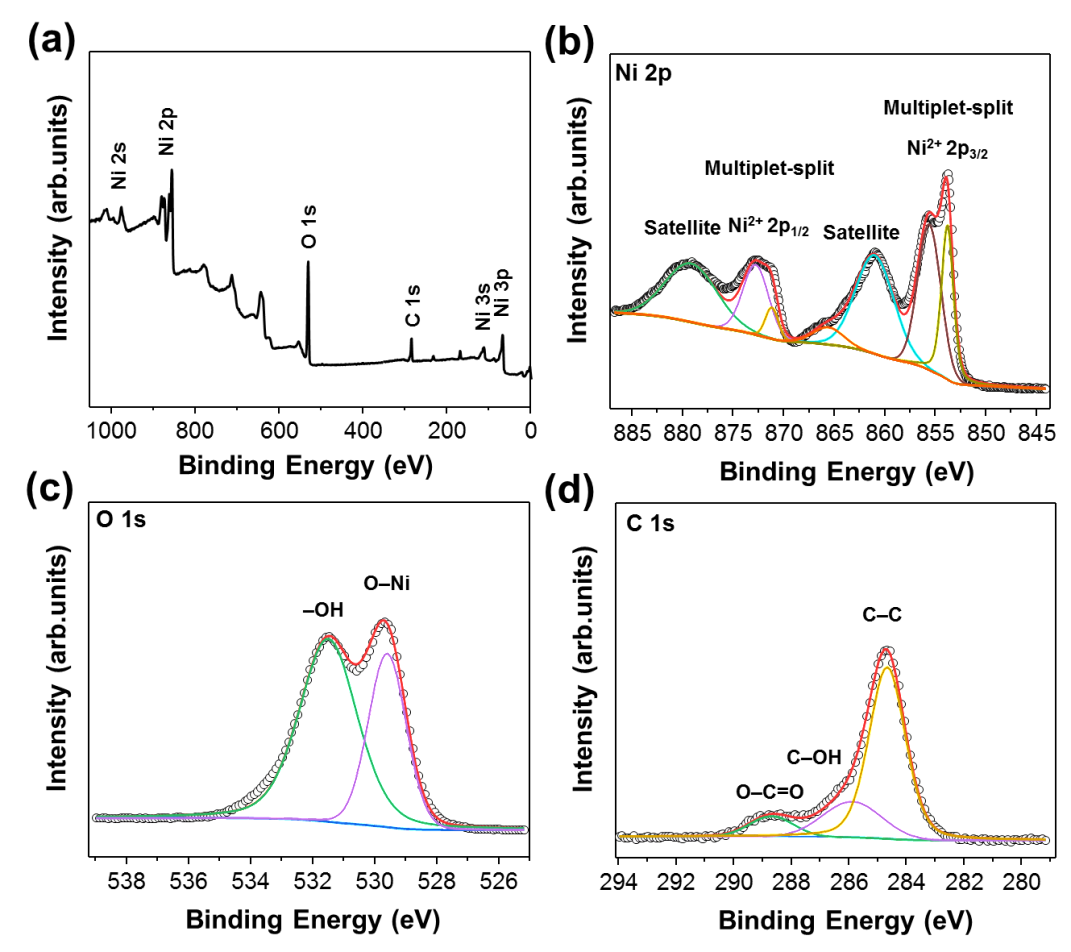


**Fig. S4**. (a) XPS survey spectrum. High-resolution XPS profiles of H-NiO/C: (b) Ni 2p, (c) O 1s, and (d) C 1s.

**
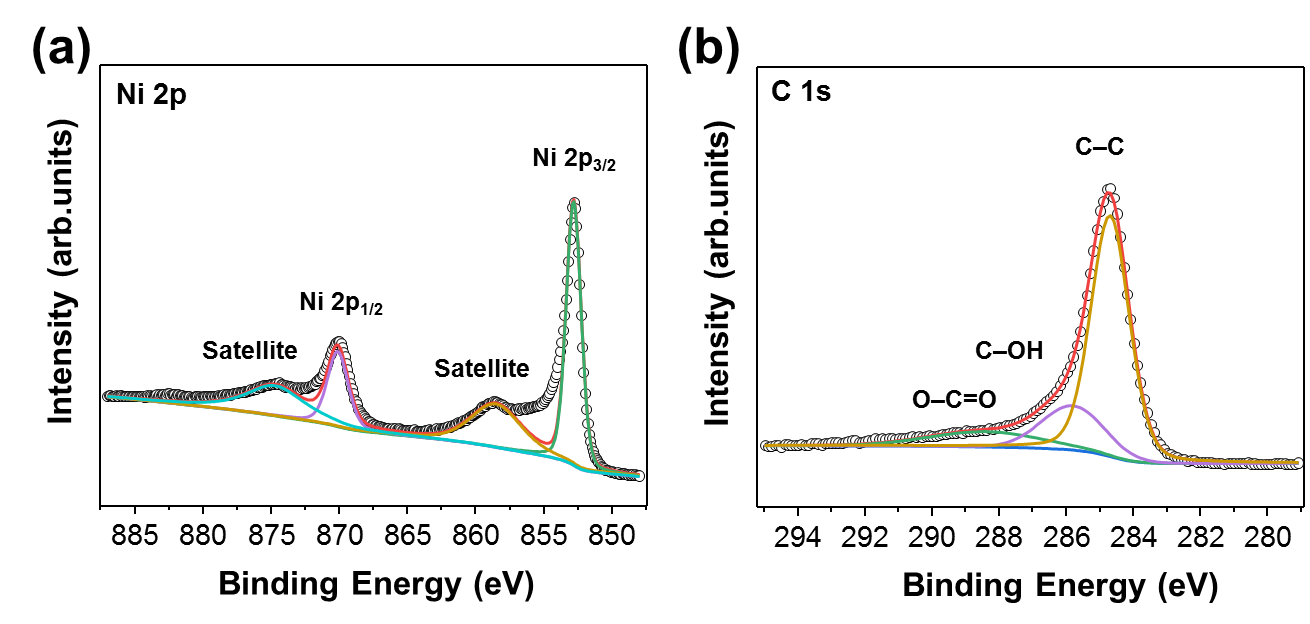
**

**Fig. S5**. High-resolution XPS profiles of H-Ni/C: (a) Ni 2p, and (b) C 1s.


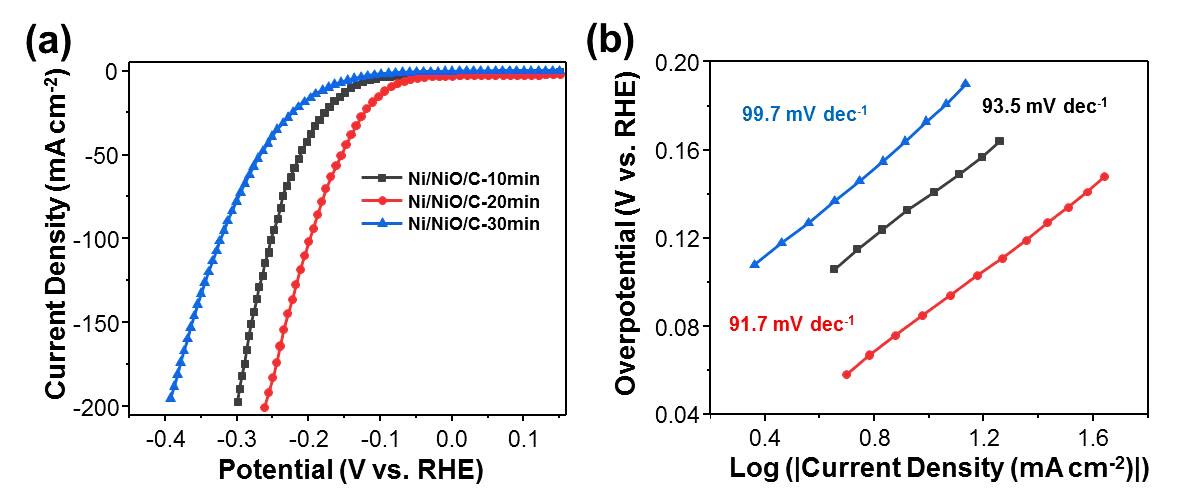


**Fig. S6**. (a) Polarization curves of the H-Ni/NiO/C-10min, H-Ni/NiO/C-20min, and H-Ni/NiO/C-30min catalysts at a scan rate of 2 mV s^-1^ in a 1 M KOH solution. (b) Tafel plots of the same catalysts derived from (a).


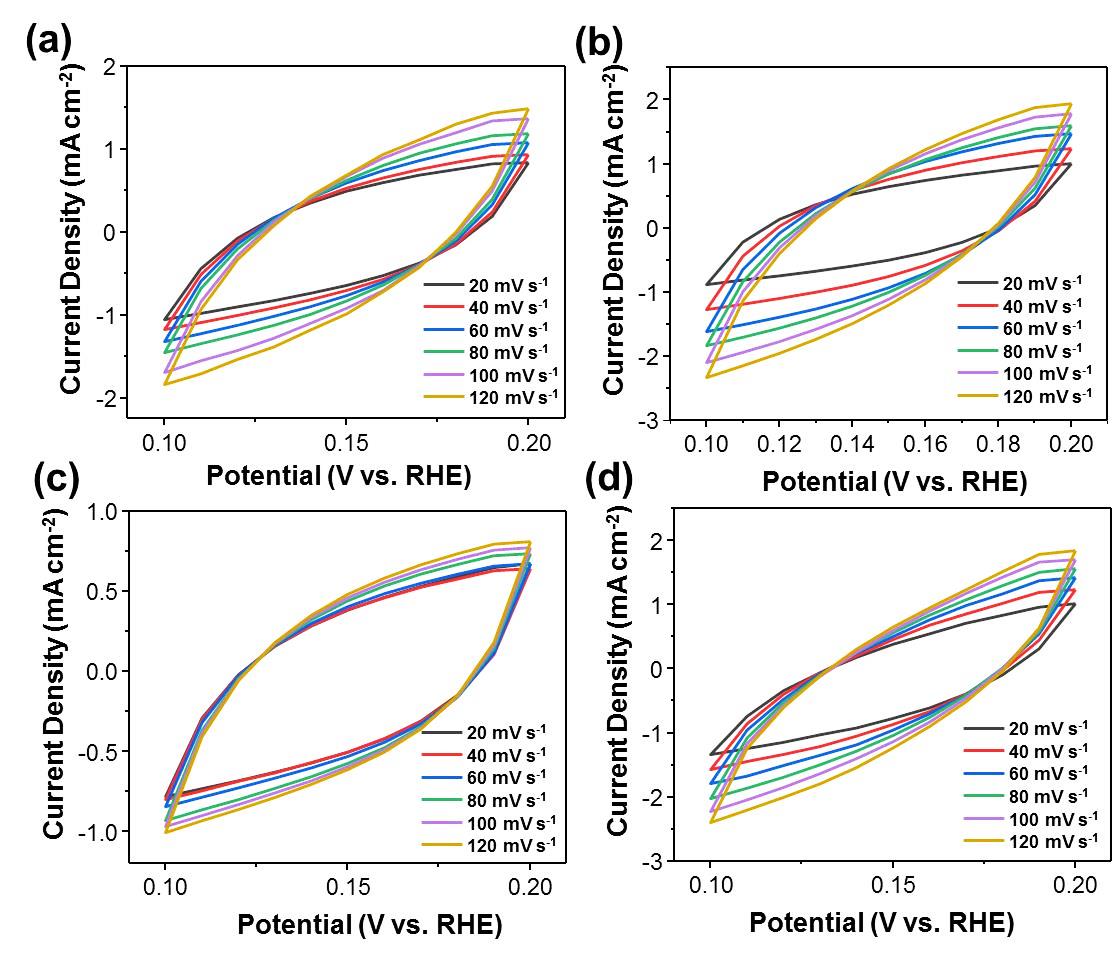


**Fig. S7**. Cyclic voltammograms (0.1–0.2 V) of (a) H-Ni/C, (b) H-Ni/NiO/C, (c) H-NiO/C, and (d) NH-Ni/NiO/C at various scan rates (20–120 mV s^-1^) in a 1 M KOH solution.


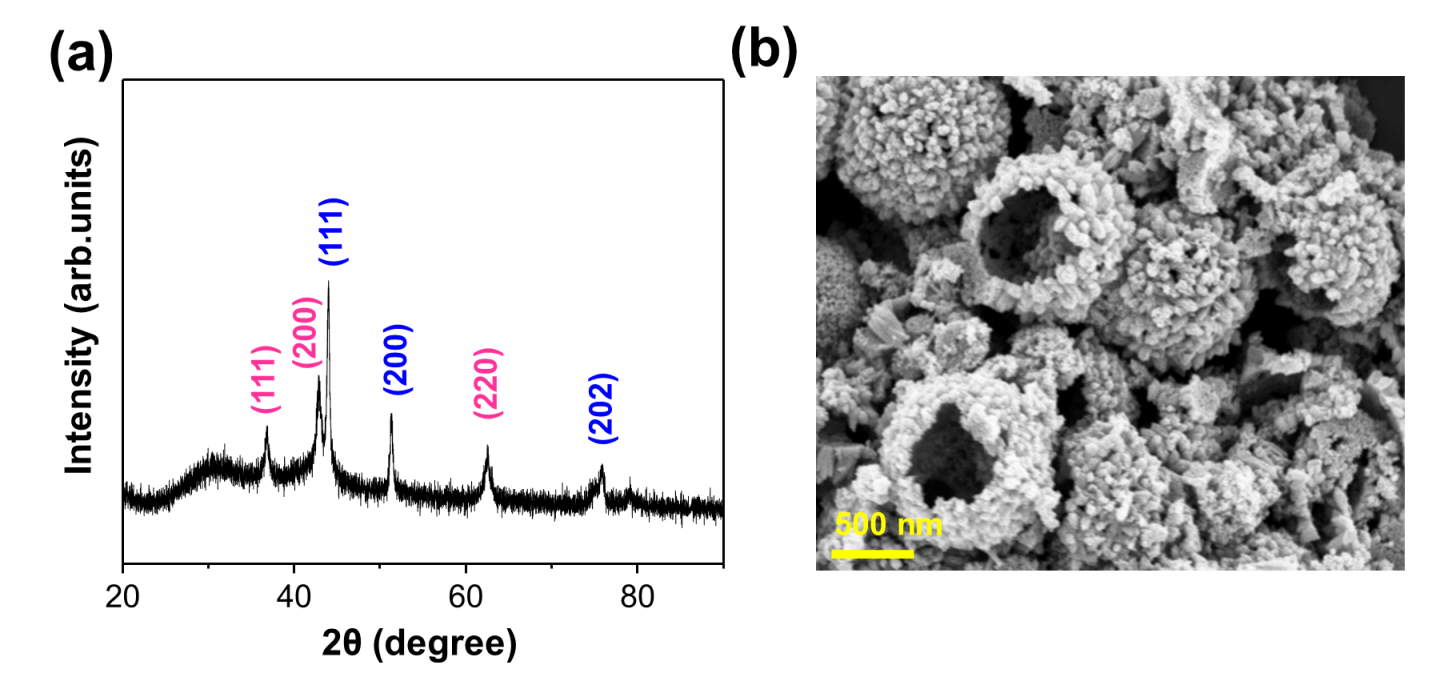


**Fig. S8**. (a) XRD pattern and (b) SEM images of H-Ni/NiO/C after 2000 CV cycles.

The ECSA was calculated using C_dl_ with the following relation:

$ECSA=\frac{C_{\mathrm{dl}}}{\mathrm{Cs}\mathrm{cm}_{\mathrm{ECSA}}^{-2}}$. (1)

where Cs = 0.040 mF cm^-2^ in an alkaline solution. The C_dl_ values were calculated from the CV profiles at different scan rates (ν) in the non-Faradaic region (from 0.1 to 0.2 V vs. the RHE), using the slope of the plot of ΔJ/2 versus the scan rate.

**Table S1:** Comparison of the HER performance of different samples in an alkaline solution

| Samples | Overpotential (mV) at 10 mA cm^-2^ | Tafel slope (mV dec^-1^) | C_dl_ (mF cm^-2^) | ECSA  (cm^2^) | R_ct_/R_ad_ (Ω) |
| --- | --- | --- | --- | --- | --- |
| H-Ni/NiO/C | 87 | 91.7 | 4.89 | 122.25 | 75.9/6.1 |
| NH-Ni/NiO/C | 117 | 98.7 | 3.69 | 92.25 | 133.4/7.4 |
| H-Ni/C | 167 | 106.5 | 2.75 | 68.75 | 189.6/8.9 |
| H-NiO/C | 246 | 124.2 | 1.08 | 27 | 229.4/14.25 |

**Table S2.** Comparison of the catalytic activity of the Ni/NiO/C hollow structure with those of nickel-based catalysts for the HER in an alkaline solution.

| Precursors | Catalysts | Current density  (mA cm^-2^) | Overpotential (mV) | Tafel slope (mV dec^-1^) | Reference |
| --- | --- | --- | --- | --- | --- |
| Ni(NO_3_)_2_·6H_2_O | Ni/NiO/NCW-1 | 10 | 105.3 | 55.2 | [[1](#_ENREF_1)] |
| NiAc/PVP | Ni/NiO-CNTs | 10 | 98 | 79 | [[2](#_ENREF_2)] |
| Ni(NO_3_)_2_·6H_2_O | NiO/C composite | 10 | 565 | 77.8 | [[3](#_ENREF_3)] |
| Ni(NO_3_)_2_·6H_2_O | NiO | 5 | 209 | 60 | [[4](#_ENREF_4)] |
| Ni metal | NiO/Ni@C-O | 10 | 89 | - | [[5](#_ENREF_5)] |
| Ni(OH)_2_ | Ni/NiO | 10 | 120 | 114 | [[6](#_ENREF_6)] |
| **Ni-MOF** | **H-Ni/NiO/C** | **10** | **87** | **91.7** | **This work** |

**References**

[1] H. Han, S. Park, D. Jang, W.B. Kim, N-doped carbon nanoweb-supported Ni/NiO heterostructure as hybrid catalysts for hydrogen evolution reaction in an alkaline phase. J. Alloys Compd. **853,** 157338 (2021)

[2] L. Yang, X. Zhao, R. Yang, P. Zhao, Y. Li, P. Yang, J. Wang, D. Astruc, In-situ growth of carbon nanotubes on Ni/NiO nanofibers as efficient hydrogen evolution reaction catalysts in alkaline media. Appl. Surf. Sci. **491,** 294-300 (2019)

[3] S. Lu, M. Hummel, Z. Gu, Y. Gu, Z. Cen, L. Wei, Y. Zhou, C. Zhang, C. Yang, Trash to treasure: A novel chemical route to synthesis of NiO/C for hydrogen production. International Journal of Hydrogen Energy **44,** 16144-16153 (2019)

[4] X. Yan, L. Tian, X. Chen, Crystalline/amorphous Ni/NiO core/shell nanosheets as highly active electrocatalysts for hydrogen evolution reaction. J. Power Sources **300,** 336-343 (2015)

[5] J. Wang, Z. Zhao, C. Shen, H. Liu, X. Pang, M. Gao, J. Mu, F. Cao, G. Li, Ni/NiO heterostructures encapsulated in oxygen-doped graphene as multifunctional electrocatalysts for the HER, UOR and HMF oxidation reaction. Catal. Sci. Technol. **11,** 2480-2490 (2021)

[6] Y. Kuang, G. Feng, P. Li, Y. Bi, Y. Li, X. Sun, Single‐crystalline ultrathin nickel nanosheets array from in situ topotactic reduction for active and stable electrocatalysis. Angew. Chem. **128,** 703-707 (2016)
